# Feasibility and Considerations for Remote Interviewing of Persons Living With Dementia: Adaptations for COVID-19

**DOI:** 10.1093/geroni/igab046.983

**Published:** 2021-12-17

**Authors:** Jeanine Yonashiro-Cho, Elizabeth Avent, Laura Mosqueda, Zachary Gassoumis

**Affiliations:** 1 University of Southern California, Alhambra, California, United States; 2 University of Southern California, Los Angeles, California, United States; 3 Keck School of Medicine of USC, Alhambra, California, United States

## Abstract

The continuing COVID-19 pandemic has necessitated changes to research protocols and approaches to mitigate health risks to both study participants and researchers. This is particularly true of studies exploring the biopsychosocial well-being and personal perspectives of older adults and those at elevated risk of COVID-19 complications. While videoconferencing platforms have enabled remote work and social activities, reliance on them may potentially exclude some individuals (e.g., those without digital devices, access to high speed internet or proficiency with technology). Persons living with dementia (PLWD) may experience difficulties navigating videoconferencing systems and building rapport with interviewers, though the inclusion of PLWD in research is necessary to ensuring their equitable representation. This presentation disseminates promising practices and lessons learned from a longitudinal study conducting remote interviews on sensitive topics with PLWD and their care partners (CP). Findings are drawn from a case study of the Better Together Dementia Care Study, an 18-month longitudinal study of PLWD (N=8) and their CPs (N=13), which implemented remote interviewing in Summer 2020 to gather data on the quality-of-life, resilience, relationship quality, adverse childhood experiences, mistreatment, and health status of PLWD. Researchers were able to interview most enrolled PLWD (n=7) via videoconferencing. Paper surveys were mailed to phone-interviewed participants, enabling them to view questions and answer choices in concordance with verbal queries. Researchers also tested a protocol asking CPs to leave the room while PLWD answered questions on sensitive topics. Findings support the use of remote interviewing with PLWD and provide insights to guide replication of these approaches.

